# Laboratory Abnormalities in Acne Patients Treated With Oral Isotretinoin: A Retrospective Epidemiological Study

**DOI:** 10.7759/cureus.19031

**Published:** 2021-10-25

**Authors:** Abdullah Alajaji, Faisal A Alrawaf, Saleh I Alosayli, Hana N Alqifari, Bader M Alhabdan, Majed A Alnasser

**Affiliations:** 1 Department of Dermatology, Qassim University College of Medicine, Buraidah, SAU; 2 Department of Statistics, Qassim University College of Science, Buraidah, SAU

**Keywords:** cholesterol, triglycerides, liver enzymes, isotretinoin, acne vulgaris

## Abstract

Background

Isotretinoin has been used to treat moderate to severe acne. It is well known that isotretinoin can cause an elevation in liver enzymes, triglycerides, and cholesterol. Laboratory monitoring is indicated while patients are on isotretinoin, but the frequency of laboratory monitoring is very variable among physicians who prescribe it.

Study objectives

This study aimed to determine the frequency of laboratory abnormalities of triglycerides, cholesterol, and liver aminotransferases in acne patients treated with oral isotretinoin in order to assess the need for frequent laboratory monitoring while on isotretinoin and to study the association between body weight and laboratory abnormalities.

Methods

A retrospective chart review has been conducted using data extracted from electronic medical records of the Department of Dermatology, Qassim University Medical City, Saudi Arabia. We included all acne patients who were treated with Isotretinoin for at least four months. Data were analyzed using the statistical program SPSS version 25 (Armonk, NY: IBM Corp.).

Results

A total of 407 patients met the inclusion criteria and were included in our study, 198 (48.6%) were female and 209 (51.4%) were males. Patients' age ranged from 10 to 51 years, with a mean age of 22.15 years. At baseline, aspartate aminotransferase (AST) was elevated in 5.4% of patients and alanine aminotransferase (ALT) was elevated in 12.7% of patients. At the last visit, AST was elevated in 3.9% of patients while ALT was elevated in 9% of patients. Triglycerides level was elevated in 12.7% of patients at the last visit compared to 6.5% of patients at baseline. Total cholesterol was elevated in 9% of patients at the last visit compared to 10.5% of patients at baseline. The increase in triglyceride levels and differences between triglycerides (TG) classifications between baseline and last visit was statistically significant (P<0.001). Higher body weight was associated with a higher incidence of elevation in ALT and triglycerides levels, and this association was statistically significant. There was no statistically significant relationship between total cumulative dose and laboratory abnormalities in ALT, AST, triglycerides, or total cholesterol.

Conclusion

The findings of this study indicate that oral isotretinoin can cause an elevation in ALT, AST, total cholesterol, and triglyceride levels but the incidence of these laboratory abnormalities is low and the elevation was not associated with significant morbidity, and therefore the practice of monthly laboratory monitoring for all patients while on isotretinoin needs to be revised as there is no strong evidence for such practice. We also found that patients with higher body weight are at higher risk of laboratory abnormalities and may require more frequent laboratory monitoring. Our findings support less frequent laboratory monitoring for acne patients on isotretinoin who had normal baseline labs. Frequent laboratory monitoring in these patients carries financial and emotional implications and lacks strong evidence to support this practice.

## Introduction

Acne is a very common skin condition. It is considered a follicular disease that has two stages, comedonal and inflammatory stage [1]. A comedon is formed when faulty keratinization results in a hyperkeratotic plug that blocks the opening of the pilosebaceous cell. Seborrhea (excess sebum production) causes follicular dilatation which contributes to bacterial overgrowth of *Propionibacterium acne*, a follicle colonizer that can cause leukocyte infiltration and follicular rupture [2]. Inflammation is triggered by follicular contents and bacterial metabolites, resulting in papules and pustules. Androgens, especially dehydroepiandrosterone sulfate (DHEAS) also contribute to acne formation. Scarring and post-inflammatory hyperpigmentation are typical residual skin changes [2]. Acne vulgaris affects 79-95% of the teenage population [3]. Acne affects predominantly youth, but it can also affect children and adults [4].

Given aesthetic reasons, acne has a negative psychosocial effect on patients especially teenage girls, and it also can affect the quality of life [5]. Acne patients are more likely to suffer from depression, anxiety, and other psychological disorders and acne treatment can help to alleviate these symptoms [6].

Acne treatment is decided by the severity of the disease [7]. In cases of non-inflammatory acne or mild inflammatory acne, topical tretinoin, adapalene, benzoyl peroxide, azelaic acid, and topical antibiotics are commonly used, either alone or in combination. Isotretinoin is indicated for the treatment of moderate to severe inflammatory acne as well as cases of acne that are resistant to other treatment options including antibiotics and topical agents [8]. Isotretinoin doses in these cases range from 0.5 to 2 mg/kg per day, given over a 16 to 24-week period. The usual recommended cumulative dose is 120-150 mg/kg [7]. Isotretinoin is a vitamin A-derived drug that has a powerful anti-sebum effect that reduces sebaceous gland activity and size, normalizes sebaceous follicle keratinization, and reduces the number of *Propionibacterium acnes* [8].

Isotretinoin has known side effects and can cause laboratory changes including elevation in liver enzymes, triglycerides (TG), and total cholesterol [7]. The most serious side effect is teratogenicity, but the most common mucocutaneous side effects are dry cracked lips, dryness of skin, and nasal mucosa. 

The incidence of isotretinoin-associated laboratory abnormalities varies between different studies, and there is a wide variation in the frequency of laboratory monitoring among different prescribing physicians. The practice of monthly laboratory monitoring among isotretinoin-treated patients lacks evidence and our objective was to address this issue by studying the incidence of these laboratory abnormalities in order to see if frequent monitoring carries a large impact on clinical management on such patients or not. In addition, we studied the effect of body weight and total cumulative dose on laboratory abnormalities.

## Materials and methods

We conducted our retrospective study at the Department of Dermatology, Qassim University Medical City. Ethical approval was obtained before starting this study from Qassim University Research Ethics Committee (#200805) on April 15, 2021.

We included all patients who received oral isotretinoin for the treatment of acne vulgaris between June 2016 and February 2021 who have completed a minimum of two isotretinoin laboratory tests during the treatment course, and these tests include aspartate aminotransferase (AST), alanine aminotransferase (ALT), triglycerides (TG), and total cholesterol (TC). Data obtained from patient medical records included age, sex, duration of treatment, starting dose, dose at the end of the treatment, baseline labs, follow-up labs, and labs from the last visit. Statistical analysis was performed using SPSS version 25 (Armonk, NY: IBM Corp.). We reviewed laboratory results of all patients and compared labs at baseline to follow-up labs and labs at the end of the treatment course. We also studied the relationship between total cumulative dose and laboratory abnormalities. The relationship between body weight and laboratory changes during the course of treatment was also studied.

The total number of patients diagnosed with acne vulgaris was 2304. Patients who did not receive oral isotretinoin were excluded (n=1498). There were 858 patients who received oral isotretinoin but those who did not meet the inclusion criteria were excluded (n=451). Thus, in the end, 407 patients were included in the study.

In this study, we evaluated changes in AST, ALT, TG, and TC levels in 407 acne patients who were treated with oral isotretinoin. Analysis of AST, ALT, TG, and TC levels was based on the National Cholesterol Education Program guidelines [9]. Aspartate aminotransferase and ALT levels were classified as normal (<40 U/L) and high (≥40 U/L) [2]. Triglyceride (TG) level was classified as normal (≤150 mg/dL) and high (>150 mg/dL), where total cholesterol (TC) was classified as normal (200 mg/dL) and high (>200 mg/dL).

Data analysis

Repeated measures analysis of variance was performed to compare means of laboratory values at three different intervals (baseline, follow-up, and last visit values). We used this test to study the statistical difference between the above three interval tests (baseline, follow-up, and last visit values). A paired sample t-test was used to compare means between any two groups. All tests were two-sided, with type 1 error rate of 0.05 thus the results with a p-value less than 0.05 were considered statistically significant. The analysis was done with SPSS version 25.

## Results

Of the 407 patients included in our study, 198 (48.6%) were female and 209 (51.4%) were males (Table 1). Patients' age ranged from 10 to 51 years, with mean age of 22.15 years. The mean and (standard deviation /SD) age of the women was 22.2 (4.2) years and 22.1 (3.9) years for men (Table 1).

**Table 1:** Demographic characteristics of the acne patients who had been treated with oral isotretinoin (n=407). SEM: standard error of the mean

Demographic characteristics		Mean (SEM)
Age (years)		22.15 (0.2)
Gender	Men	209 (51.4%)
	Women	198 (48.6%)
Body weight (kg)		66.5 (0.9)
Cumulative dose (mg)		6741.94 (253.7)
Starting dose		34.20 (0.5)
Dose at end		37.44 (0.4)

The study evaluates the effect of isotretinoin on liver enzymes (AST and ALT) and lipids (TC and TGs). Nearly all patients (94.6%) had normal AST and 87.7% of them had normal ALT levels at baseline. The results are outlined in Tables 2, 3. For a few patients, there were missing individual labs in following up or last visit labs as indicated in Table 2.

**Table 2:** Laboratory results of study patients (N=407). Some values were not recorded for all patients at each follow-up. *Statistically significant AST: aspartate aminotransferase; ALT: alanine aminotransferase; TC: total cholesterol; TG: triglyceride

Summary of laboratory results					
Laboratory value	Patients, n (%)				
	Baseline	Follow up	Last visit	F score (F_2_)	P-values
AST: normal (<40 U/L) vs high (≥40 U/L)	368 (94.6%) vs 21 (5.4%)	209 (94.6%) vs 12 (5.4%)	367 (96.1%) vs 15 (3.9%)	1.160	0.315
ALT: normal (<40 U/L) vs high (≥40 U/L)	341 (87.7%) vs 48 (12.3%)	194 (88.6%) vs 25 (11.4%)	342 (91.0%) vs 34 (9.0%)	0.904	0.407
TC: normal (≤200 mg/dL) vs high (>200 mg/dL)	357 (89.5%) vs 42 (10.5%)	194 (88.6%) vs 25 (11.4)	342 (91%) vs 34 (9%)	41.855	≤0.000*
TG: normal (≤150 mg/dL) vs high (>150 mg/dL)	373 (93.5%) vs 26 (6.5%)	185 (83.7%) vs 36 (16.3)	345 (87.3%) vs 50 (12.7%)	20.412	≤0.000*
TG: normal (<150 mg/dL) vs borderline high (150-199 mg/dL) vs high (200-499 mg/dL) vs very high (≥500 mg/dL)	373 (93.5%) vs 18 (4.5%) vs 8 (2.0%) vs 0 (0%)	184 (83.3%) vs 23 (10.4%) vs 13 (5.9%) vs 1 (0.5%)	343 (86.8%) vs 36 (9.1%) vs 16 (4.1%) vs 0 (0%)		

**Table 3:** Paired samples t-test comparing baseline laboratory result to last visit result. *Statistically significant. AST: aspartate aminotransferase; ALT: alanine aminotransferase; TC: total cholesterol; TG: triglyceride

	Paired sample t-test	df	P-value
AST: baseline-last visit	-1.726	364	0.085
ALT: baseline-last visit	1.559	359	0.120
TC: baseline-last visit	-12.047	378	0.000*
TG: baseline-last visit	-6.369	387	0.000*

Aspartate aminotransferase (AST) levels were classified as normal or high. At baseline, mean (SD) AST levels were 23.8 (13.9) U/L, with normal levels in 368 (94.6%) patients and high in 21 (5.4%) patients. At follow-up, mean (SD) AST levels were 25.5 (9.2) U/L, with normal levels in 209 (94.6%) patients and high levels in 12 (5.4%) patients. At the last visit, mean (SD) AST levels were 24.9 (11.2) U/L, with normal levels in 367 (96.1%) patients and high levels in 15 (3.9%) patients. Aspartate aminotransferase level increased at the last visit compared to baseline and follow-up but despite this increase in values, most patients remained in the normal range during the course of treatment. Differences between AST classifications (high vs normal) at the three-time points were not statistically significant (F_2_=1.160, df=2, P=0.315). Differences between AST levels at baseline compared to follow-up and last visit and differences between AST levels at follow-up and last visit were both not statistically significant with p-value of 0.181, 0.085, 0.818, respectively. Overall, the results indicated that AST levels slightly increased over time in patients treated with isotretinoin, but the increase was not above the normal range and was not statistically significant.

Alanine aminotransferase (ALT) levels were classified as normal or high. At baseline, mean (SD) ALT level was 25.5 (15.8) U/L, with normal levels in 341 (87.7%) patients and high in 48 (12.3%) patients. At follow-up, mean (SD) ALT level was 25.4 (12.9) U/L, with normal levels in 194 (88.6%) patients and high in 25 (11.4%) patients. At the last visit, mean (SD) ALT level was 23.7 (16.8) U/L, with normal levels in 342 (91.0%) patients and high in 34 (9.0%) patients. Alanine aminotransferase levels increased at follow-up and last visit compared to baseline but despite this increase in ALT levels, it was within the normal range. Overall, ALT levels increased with time, but the differences between baseline, follow-up, and last visit were not statistically significant (F_2_=0.904, df=2, P=0.407). The pairwise differences between ALT levels at baseline and follow-up, baseline and last visit, and follow-up and last visit were not statistically significant with p-value of 0.567, 0.120, 0.245, respectively. Overall, the results indicated that ALT levels increased over time in patients treated with isotretinoin but the increase was not statistically significant.

Total cholesterol (TC) levels were classified as normal or high. At baseline, mean (SD) TC levels were 162.4 (30.8) mg/dL, with normal levels in 357 (89.5%) patients and high in 42 (10.5%) patients. At follow-up, mean (SD) TC levels were 174.5 (31.6) mg/dL, with normal levels in 194 (88.6%) patients and high in 25 (11.4%) patients. At the last visit, mean (SD) TC levels were 176.9 (32.3) mg/dL, with normal levels in 342 (91%) patients and high in 34 (9%) patients. Baseline TC levels increased compared to follow-up and this was statistically significant (P< 0.000). Differences in TC levels last visit versus baseline were statistically significant (P<0.000). However, changes in TC levels from follow-up compared to last visit were not statistically significant (P=0.324). The difference between TC classifications at each time point was statistically significant (F_2_=41.855, df=2, P<0.000). Overall, TC levels at follow-up were higher than baseline, and this increase in TC levels was above the normal range.

Triglycerides (TG) levels were classified as normal or high. At baseline, mean (SD) TG level was 80.7 (30.8) mg/dL, with normal levels in 373 (93.5%) patients and high in 26 (6.5%) patients. At follow-up, mean (SD) TG levels were 101.6 (67.1) mg/dL, with normal levels in 185 (83.7%) patients and high in 36 (16.3%) patients. At last visit, mean (SD) TG levels were 96.1 (51.4) mg/dL, with normal levels in 345 (87.3%) patients and high in 50 (12.7%) patients. Triglyceride levels increased and differences between TG classifications at each time point (baseline, follow-up, and last visit) were statistically significant (F_2_=20.412, df=2, P<0.000). TG levels increased at follow-up and last visit compared to baseline TG levels and this increase was statistically significant (P=0.000). However, changes in TG levels from follow-up compared to the last visit were not statistically significant (P=0.404). Overall, TG levels increased from baseline during isotretinoin treatment at follow-up and last visit, and these increases were above the normal range.

**Figure 1:**
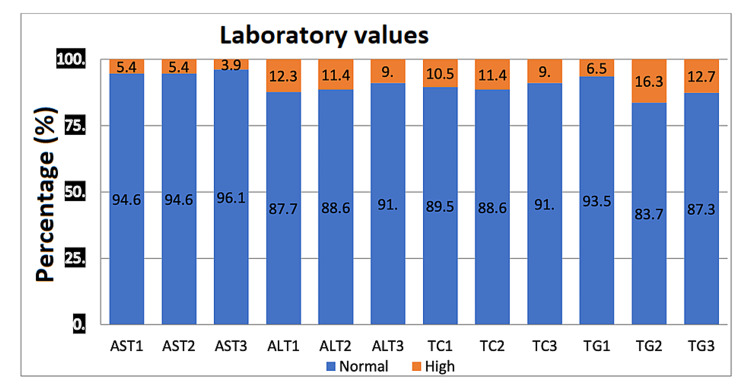
Distribution of normal and abnormal laboratory values by the percentage of patients included in the study. X-axis: AST1, AST2, AST3 - levels of aspartate aminotransferase at visit 1, 2, and 3 (normal vs high). ALT1, ALT2, ALT3 - levels of alanine aminotransferase at visit 1, 2, and 3 (normal vs high). TC1, TC2, TC3 - levels of total cholesterol at visit 1, 2, and 3 (normal vs high). TG1, TG2, TG3 - levels of triglycerides at visit 1, 2, and 3 (normal vs high). Y-axis: percentage of normal vs high level of laboratory tests among patients included in the study. AST: aspartate aminotransferase; ALT: alanine aminotransferase; TC: total cholesterol; TG: triglyceride

There was no significant relationship between body weight (BW) and AST levels at follow-up and last visit (Table 4). The difference in ALT levels at follow-up and last visit versus BW at baseline was statistically significant (P=0.030 and P=0.001). TC levels compared to BW were not statistically significant for follow-up and last visit levels with p-value of 0.107, 0.628, and 0.946, respectively. TG levels compared to BW were statistically significant for follow-up and last visit levels with p-value ≤0.005 and ≤0.001 (Table 5). There was no statistically significant relationship found between cumulative dose (CD) and AST, ALT, TC, and TG levels (Table 6). The summary of laboratory results is shown in Figure 1.

**Table 4:** Mean values and standard deviation of body weight (BW) of patients in relation to laboratory abnormalities. AST: aspartate aminotransferase; ALT: alanine aminotransferase; TC: total cholesterol; TG: triglyceride

	Mean of BW		SD BW	
Mean and (SD) of patients	Normal level	High level	Normal level	High level
BW for AST baseline levels	66.1	77.3	16.6	22.0
BW and AST last visit levels	66.2	75.8	16.9	22.7
BW and ALT baseline levels	65.5	76.1	16.6	18.2
BW and ALT last visit levels	65.5	76.6	16.9	18.0
BW and TC baseline levels	66.1	70.9	16.9	17.3
BW and TC last visit levels	66.3	66.1	17.4	15.0
BW and TG baseline levels	65.8	81.4	16.5	19.3
BW and TG last visit levels	64.8	77.9	16.5	16.1

**Table 5:** Independent samples t-test to test the difference in the BW and AST, ALT, TC, and TG levels. *Statistically significant. AST: aspartate aminotransferase; ALT: alanine aminotransferase; TC: total cholesterol; TG: triglyceride

	Independent samples t-test	df	P-value
BW of AST last visit levels	-1.906	319	0.058
BW of ALT last visit levels	-3.378	314	0.001*
BW of TC last visit levels	0.068	324	0.946
BW of TG last visit levels	-4.896	329	0.000*

**Table 6:** Independent samples t-test to test the relationship between cumulative dose (CD) and AST, ALT, TC, and TG levels in the last visit results. *Statistically significant. AST: aspartate aminotransferase; ALT: alanine aminotransferase; TC: total cholesterol; TG: triglyceride

	Independent samples t-test	df	P-value
CD of AST last visit levels	1.196	376	0.232
CD of ALT last visit levels	-0.240	370	0.810
CD of TC last visit levels	0.833	380	0.405
CD of TG last visit levels	0.746	389	0.456

## Discussion

We found that the incidence of abnormal elevation in ALT and AST in patients with acne treated with isotretinoin is low and was not associated with significant morbidity. Total cholesterol and triglycerides levels increased during the treatment and this was statistically significant but the increase in levels was mild. We found that patients with higher body weight are at higher risk of elevations in ALT and triglycerides levels at the end of the treatment course. The frequency of laboratory monitoring for patients on isotretinoin varies between dermatologists. Many dermatologists request monthly laboratory monitoring for their acne patients on isotretinoin.

Our study result support less frequent laboratory monitoring for healthy patients with normal baseline labs. Our findings confirmed data from other recent studies that have shown evidence that frequent monitoring of labs while on isotretinoin is not necessary for those with normal baseline labs. Shah and Kroshinsky reviewed 903 patients who were diagnosed with acne vulgaris and treated with isotretinoin therapy from 2010 to 2017, and their findings support the thought that transient alterations of lipid and liver aminotransferases levels tend to be clinically insignificant and monthly laboratory monitoring may be unnecessary [10]. A recent meta-analysis published by Lee et al. that included 1574 patients from 26 studies concluded that there is no evidence to support monthly laboratory testing for standard acne patients on standard isotretinoin dosing [11]. This meta-analysis showed that isotretinoin is associated with a statistically significant change in the mean value of several laboratory tests (white blood cell count and hepatic and lipid panels), yet high-risk or severe abnormalities are infrequent. Barbieri et al. reviewed 1863 patients treated with isotretinoin and found that grade 3 or greater triglyceride and liver function testing abnormalities were noted in fewer than 1% and 0.5% of patients screened, respectively, and authors recommended less frequent monitoring of lipid and liver function tests [12]. In a retrospective study, Hansen et al. reviewed 515 patients treated with isotretinoin and the study showed that elevation in liver transaminases was infrequent and levels were not increased significantly compared with baseline rates (1.9% vs 1.6% at baseline) but there was significant elevations in triglyceride (19.3%) and cholesterol (22.8%) levels but the elevations were mild to moderate, and based on this study result, the authors recommended that in healthy patients on isotretinoin, lipid panel and liver function test be performed at baseline and second month, and further testing should be considered only if a significant abnormality in laboratory tests is noted [13]. In a recent study, Sharma and Tkachenko reviewed 735 patients and recommended less frequent laboratory monitoring for isotretinoin in healthy patients [14]. A similar conclusion was reached by Öktem et al. after reviewing 705 patients on isotretinoin [15].

Limitations of this study include the retrospective nature of this study and the fact that it is a single-center study. For few patients, there were missing individual labs from follow-up or last visit labs, and this was indicated in the tables.

## Conclusions

In conclusion, our study confirms findings from previous studies that oral isotretinoin can cause an elevation in ALT, AST, total cholesterol, and triglyceride levels. But we found that the incidence of these laboratory abnormalities is low and the elevation was not associated with significant morbidity. We also found that patients with higher body weight are at higher risk of laboratory abnormalities and may require more frequent laboratory monitoring. Our findings support less frequent laboratory monitoring for acne patients on isotretinoin who are healthy and have normal baseline labs. In these patients, especially if the dose is not increased, frequent laboratory monitoring carries financial and emotional implications and lacks strong evidence that support this practice.
